# Plasma Sphingosine-1-Phosphate Is Elevated in Obesity

**DOI:** 10.1371/journal.pone.0072449

**Published:** 2013-09-06

**Authors:** Greg M. Kowalski, Andrew L. Carey, Ahrathy Selathurai, Bronwyn A. Kingwell, Clinton R. Bruce

**Affiliations:** 1 Integrative Physiology and Metabolism Laboratory, Department of Physiology, Monash University, Clayton, Victoria, Australia; 2 Metabolic and Vascular Physiology Laboratory, Baker IDI Heart and Diabetes Institute, Melbourne, Victoria, Australia; Monash University, Australia

## Abstract

**Background:**

Dysfunctional lipid metabolism is a hallmark of obesity and insulin resistance and a risk factor for various cardiovascular and metabolic complications. In addition to the well known increase in plasma triglycerides and free fatty acids, recent work in humans and rodents has shown that obesity is associated with elevations in the bioactive class of sphingolipids known as ceramides. However, in obesity little is known about the plasma concentrations of sphinogsine-1-phosphate (S1P), the breakdown product of ceramide, which is an important signaling molecule in mammalian biology. Therefore, the purpose of this study was to examine the impact of obesity on circulating S1P concentration and its relationship with markers of glucose metabolism and insulin sensitivity.

**Methodology/Principal Findings:**

Plasma S1P levels were determined in high-fat diet (HFD)-induced and genetically obese (*ob/ob*) mice along with obese humans. Circulating S1P was elevated in both obese mouse models and in obese humans compared with lean healthy controls. Furthermore, in humans, plasma S1P positively correlated with total body fat percentage, body mass index (BMI), waist circumference, fasting insulin, HOMA-IR, HbA1c (%), total and LDL cholesterol. In addition, fasting increased plasma S1P levels in lean healthy mice.

**Conclusion:**

We show that elevations in plasma S1P are a feature of both human and rodent obesity and correlate with metabolic abnormalities such as adiposity and insulin resistance.

## Introduction

The global prevalence of obesity is growing at an alarming rate. This is of particular concern especially given that obesity is associated with an array of metabolic complications, including insulin resistance and type 2 diabetes mellitus [1]. In addition to defects in glucose metabolism, these conditions are also closely associated with dyslipidaemia, evident by an increase in circulating lipids including triglycerides and free fatty acids [2]. The dyslipidaemia is thought to arise from an oversupply of both exogenous dietary chylomicron derived lipids along with endogenously synthesized lipids such as those released from the liver (ie. cholesterol, triacylglycerol) and adipose tissue (ie. free fatty acids). Traditionally, elevations of these commonly measured circulating lipids have been used clinically to assess the risk of metabolic and heart disease [3]. While this approach has been useful, recently there has been an emergence of research attempting to identify other circulating lipid species that may prove to be more accurate biomarkers of disease onset and progression [4]–[7]. In this regard, circulating sphingolipids have emerged as potential biomarkers and mediators of disease development [8]–[12].

Sphingolipids are a complex class of lipids that play important structural, signaling and cell recognition roles [13] and have been implicated in the development of metabolic diseases including insulin resistance and type 2 diabetes [4], [8], [9], [12], [14], [15]. Plasma ceramide levels are elevated in type 2 diabetic patients [8], [14] and are inversely associated with measures of insulin sensitivity [8]. Moreover, we recently demonstrated that circulating ceramide is taken up and deposited in skeletal muscle promoting the development of insulin resistance [14]. While we are beginning to develop an appreciation of the importance of circulating ceramide [4], [8], [9], [12], [14], [15], the role of other sphingolipids, in particular sphingosine-1-phosphate (S1P), has received little attention with respect to its relationship with obesity, insulin resistance and type 2 diabetes. S1P is a breakdown product of ceramide metabolism and circulates in mammals at high levels (>200 nM), whereas in the interstitial fluid the levels are very low, thus creating an S1P compartment gradient [16], [17]. It is thought that both haematopoietic and vascular endothelial cells are the major contributors to the plasma S1P pool [16], [18], with approximately 65% of the plasma S1P being bound to lipoproteins, of which ∼50% is associated with HDL, while the remainder is bound to albumin [17], [19]. S1P has effects on many biological processes, with some of the most well characterized effects being on the immune and vascular systems, particularly in the process of inflammation [13], [20]. S1P has both physiological and pathophysiological roles, regulating cell signaling, proliferation, migration, survival, differentiation and metabolism, and its effects can be traced to essentially all organ systems [13], [20]. Interestingly, plasma S1P levels are increased in the genetically obese *ob/ob* mouse [12] and in rodent models of type 1 diabetes [21]. Furthermore, *in vitro* studies using C2C12 myoblasts have shown that S1P can stimulate basal glucose uptake by trans-phosphorylation of the insulin receptor [22], while in rat primary adipocytes S1P has been shown to stimulate lipolysis [23]. These findings suggest that S1P levels may either effect or be affected by the metabolic status of the individual. However, there is currently little known about the circulating levels of S1P in obesity and insulin resistance. Therefore, the purpose of this study was to examine the impact of obesity on circulating S1P levels and to determine whether there is a relationship between its concentrations and clinical indices of metabolic dysfunction.

## Materials and Methods

### Ethics Statement

All animal experiments were approved by the Monash University Animal Research Platform Animal Ethics Committee and were in accordance with the National Health and Medical Research Council of Australia Guidelines on Animal Experimentation. The human study was approved by The Alfred Human Research Ethics Committee, performed in accordance with the Declaration of Helsinki (fifth revision, 2000) and each participant provided written informed consent.

### Animal Studies

Male C57BL/6 mice at 8 weeks of age were fed a control chow (∼9% energy as fat, Barastok Rat & Mouse, Ridley AgriProducts, Melbourne, Australia) or high-fat diet (∼42% energy as fat, Specialty Feeds SF4-001, Glen Forrest, WA, Australia) for 6 weeks. Genetically obese male *ob/ob* mice on a pure C57BL/6 background and lean littermate controls maintained on a standard chow diet were studied at 20 weeks of age. Metabolic characteristics of the mice are presented in Table 1 and 2. Blood collection was performed after a 5 h fast via the tail vein. For the fed and fasting study, 8 week old male C57BL/6 mice on a chow diet had tail vein blood collected at 06∶00 (fed condition) after which food was removed until 18∶00 (fasted state) when blood was again collected for biochemical analysis.

**Table 1:** Metabolic characteristics of the chow and high-fat diet fed mice.

	Chow	HFD	P-Value
Sample size (N)	13	14	N/A
Body mass (g)	31.1±0.9	39.7±1.2	P<0.001
Lean mass (g)	26.8±0.9	26.8±0.6	P = 0.94
Fat mass (g)	3.1±0.4	11.9±0.8	P<0.001
Fasting blood glucose (mmol/l)	10.3±0.4	12.1±0.5	P = 0.009
Fasting plasma insulin (pmol/l)	90.0±6.1	624.1±88.7	P<0.001
Plasma NEFA (mmol/l)	0.55±0.07	0.58±0.02	P = 0.71
Plasma triglyceride (mmol/l)	0.40±0.03	0.55±0.02	P<0.001

Data are mean ± SEM. HFD; high-fat diet.

**Table 2:** Metabolic characteristics of the lean and *ob/ob* mice.

	Lean	*ob/ob*	P-Value
Sample size (N)	6	8	N/A
Body mass (g)	29.4±0.7	61.0±0.9	P<0.001
Subcutaneous fat pad (g)	0.5±0.1	4.5±0.7	P<0.001
Gonadal fat pad (g)	1.0±0.3	4.1±0.5	P<0.001
Fasting blood glucose (mmol/l)	6.7±0.3	10.1±1.2	P = 0.036

Data are mean ± SEM.

### Human Studies

Fifteen lean and 10 obese (BMI >30 kg/m^2^) otherwise healthy young men were studied. These individuals were recruited as part of a larger study which has recently been published [24]. The characteristics of all participants are presented in Table 3. Participants arrived at the laboratory following an overnight fast and the physical characteristics (height, weight, waist circumference brachial blood pressure) of each subject were measured. An 18-G cannula was inserted into an antecubital vein and a venous blood sample was obtained for measurement of HbA1c, blood glucose, plasma insulin and lipids. An oral glucose tolerance test (OGTT) was then performed. Subjects ingested a 75 g glucose load and blood was sampled at 60 and 120 min for the measurement of blood glucose. Body composition was measured using dual energy x-ray absorptiometry (DEXA).

**Table 3:** Participant characteristics.

	Lean	Obese	P-Value
Sample size (N)	15	10	N/A
Age (yr)	25.2±1.0	27.5±1.3	P = 0.073
Body mass (kg)	67.6±2.2	116.2±4.8	P<0.001
Height (cm)	176.5±2.4	177.5±1.8	P = 0.72
BMI (kg.m^−2^)	21.7±0.5	36.9±1.41	P<0.001
Waist circumference (cm)	78.4±1.4	114.5±2.6	P<0.001
Body fat (%)	18.0±1.6	44.8±1.6	P<0.001
Fasting blood glucose (mmol/l)	5.1±0.1	5.1±0.1	P = 0.99
60 min OGTT blood glucose(mmol/l)	5.9±0.4	7.3±0.5	P = 0.018
120 min OGTT blood glucose(mmol/l)	4.6±0.3	5.9±0.4	P = 0.009
Fasting plasma insulin (pmol/l)	38.6±6.7	91.5±13.4	P<0.001
HOMA-IR	1.3±0.3	3.0±0.5	P = 0.001
HbA1c (%)	5.5±0.1	5.5±0.1	P = 0.56
Systolic blood pressure (mm Hg)	117±3	119±2	P = 0.276
Diastolic blood pressure (mm Hg)	73.2±2.6	70.9±1.9	P = 0.99
Mean arterial pressure (mm Hg)	86.8±2.8	86.5±1.9	P = 0.572
Total cholesterol (mmol/l)	4.3±0.2	4.7±0.1	P = 0.13
LDL cholesterol (mmol/l)	2.6±0.2	3.1±0.2	P = 0.059
HDL cholesterol (mmol/l)	1.3±0.1	1.0±0.1	P = 0.015
Plasma triglyceride (mmol/l)	0.8±0.1	1.3±0.2	P = 0.041
Plasma NEFA (mmol/l)	0.30±0.03	0.43±0.05	P = 0.048
Plasma glycerol (mmol/l)	0.10±0.01	0.13±0.01	P = 0.087

Data are mean ± SEM.

### Biochemical Analyses

Venous blood samples obtained from the human subjects were centrifuged and plasma immediately frozen. Plasma was subsequently analyzed for total cholesterol, HDL-cholesterol, LDL-cholesterol, triacylglycerol (enzymatic methods, automated analyser), insulin (chemiluminescent microparticle immunoassay) and HbA1c (boronate-affinity HPLC) (all conducted according to clinical diagnostic standards by Alfred Hospital Pathology). For the animal studies, plasma measurements were performed on blood collected from the tail vein and centrifuged at 6000**rpm for 10 min at 4°C. Insulin was measured by ELISA (Linco Research, St. Louis, MO, USA). Plasma non-esterified fatty acids (NEFA) were determined using a colorimetric kit (Wako Pure Chemical Industries, Osaka, Japan). Free plasma glycerol levels were determined using a commercially available kit (Sigma Aldrich, St. Louis, MO, USA). Plasma S1P was measured with a commercially available competitive ELISA designed for plasma/serum determination of S1P concentration (Echelon, Salt Lake City, UT). The S1P ELISA measures total S1P concentrations in plasma/serum without distinction between lipoprotein/albumin bound S1P fractions.

### Statistical Analysis

Data are reported as mean ± SEM. Comparisons between data from two groups were made using students two-tailed un-paired or paired (fed/fasting experiments) *t*-tests. Associations between variables were investigated using Spearman correlation coefficient with GraphPad Prism software. Statistical significance was accepted at *P<0.05*.

## Results

### Plasma S1P is Elevated in Mouse Models of Obesity and Insulin Resistance

**Figure 1:**
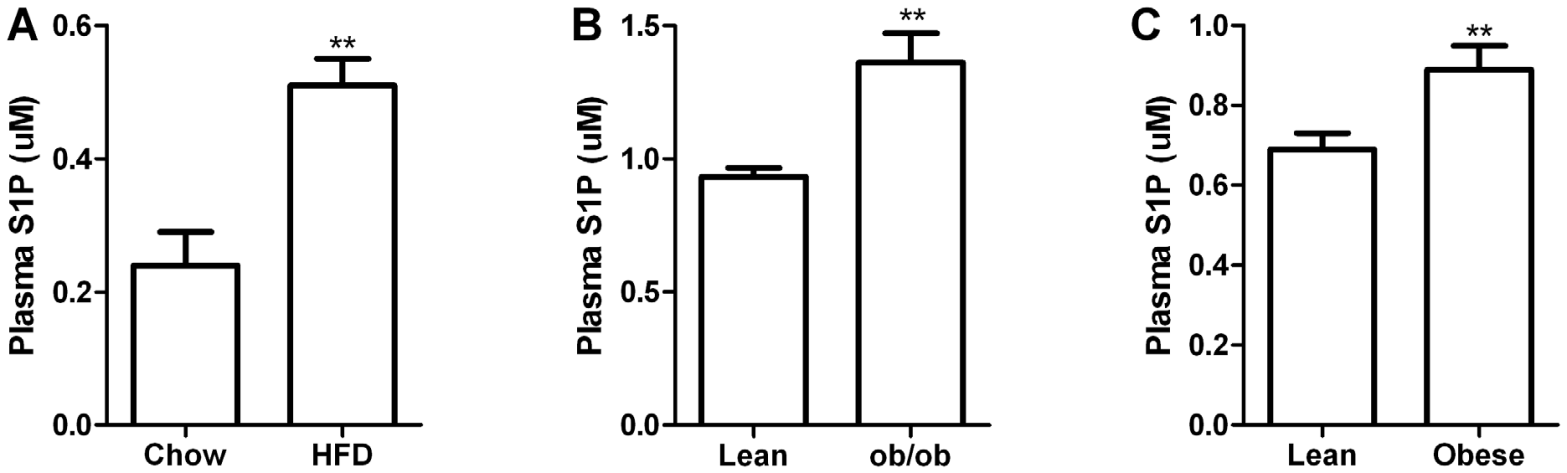
Plasma S1P concentrations are elevated in obesity. Plasma S1P concentrations in chow (N = 13) and HFD (N = 14) fed mice (A); plasma S1P levels in lean (N = 6) and *ob/ob* (N = 8) mice (B) and circulating S1P in lean (N = 15) and obese (N = 10) humans (C). Data are mean ± SEM. **P<0.01.

To determine if circulating S1P levels are altered in obesity and insulin resistance, we studied two commonly used mouse models of obesity and insulin resistance; the chronically HFD fed and genetically obese chow fed *ob/ob* mouse. The metabolic characteristics of these models are presented in Table 1 and 2. As expected, mice maintained on the HFD displayed key metabolic disturbances associated with obesity such as fasting hyperglycaemia, hyperinsulinaemia, and elevated plasma triglycerides (Table 1). Similarly, the *ob/ob* mice displayed severe obesity and fasting hyperglycaemia when compared with their lean counterparts (Table 2). Despite the differences in the aetiology of obesity in these mouse models (i.e. one consumes excess fat and sugar calories while the other over-eats a standard laboratory chow diet), both HFD induced and genetically obese *ob/ob* mice had significantly elevated plasma S1P concentrations (Fig. 1A & B).

### Plasma S1P is Elevated in Obese Humans and Correlates Positively with Indices of Metabolic Dysfunction

**Figure 2:**
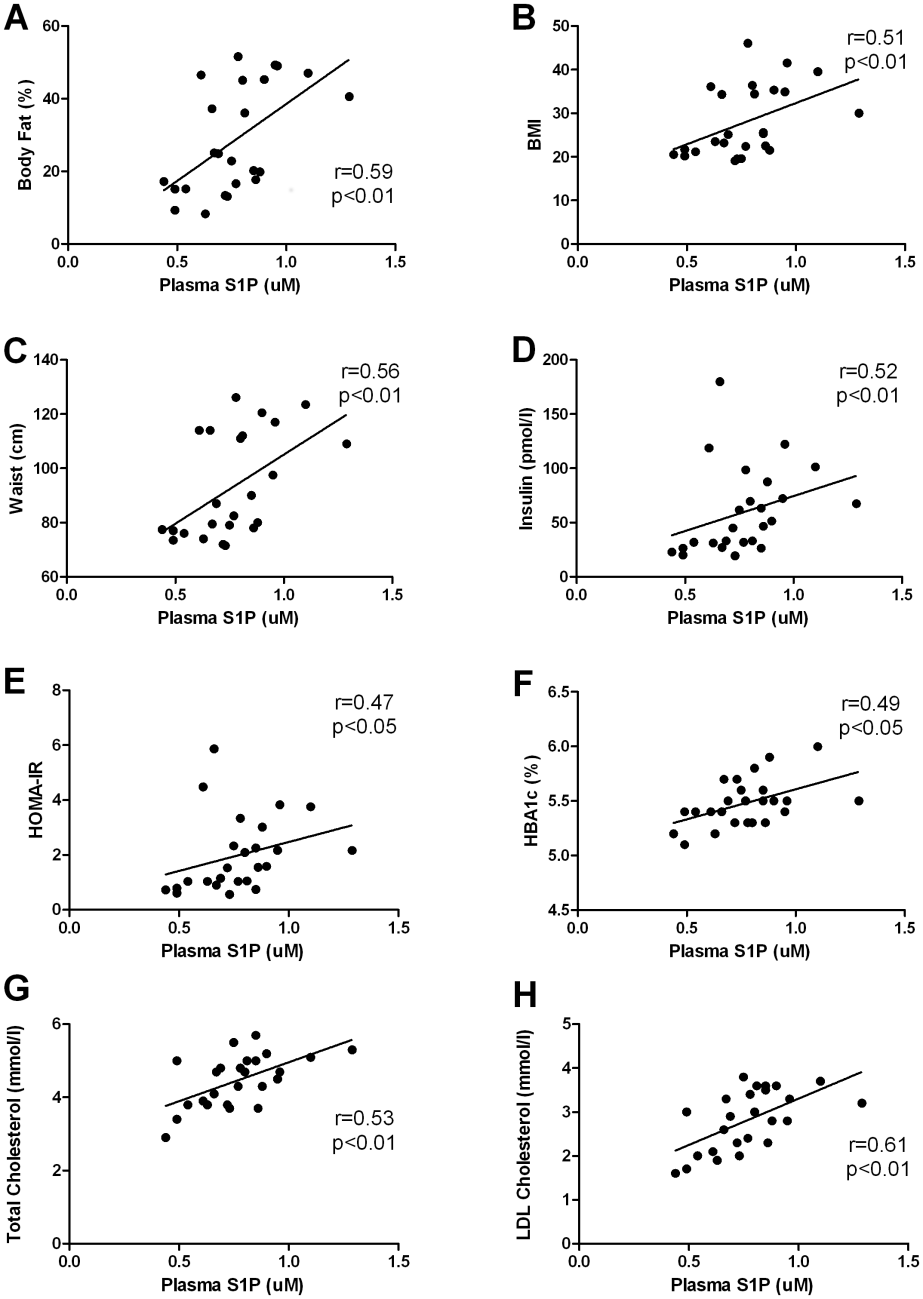
Plasma S1P concentrations correlate with clinical indices of metabolic dysfunction in humans. Plasma S1P concentrations correlate with percent body fat (A), BMI (B), waist circumference (C), fasting plasma insulin (D), HOMA-IR (E), HbA1c (F), total (G) and LDL (H) cholesterol.

To determine whether our findings of elevated plasma S1P concentrations in obese mice were also conserved in humans, we measured the plasma S1P levels in a cohort of well characterised obese (with characteristics of the metabolic syndrome) and lean control subjects. The clinical characteristics of the human subjects are presented in Table 3. Subjects did not differ in age and height and were completely un-medicated. Body mass, BMI, body fat percentage and waist circumference were higher in the obese subjects. While fasting blood glucose levels were normal in the obese individuals, fasting plasma insulin levels and HOMA-IR were elevated compared to the control subjects. Furthermore, obese subjects had significantly higher blood glucose levels at 60 and 120 min during the OGTT, indicating the presence of glucose intolerance and insulin resistance. In relation to plasma lipids, the obese subjects had significantly higher plasma triglycerides and NEFAs and significantly lower HDL cholesterol while there was a non-significant trend for LDL cholesterol (P = 0.059) to be higher in the obese subjects. Consistent with our finding in the rodent models of obesity, plasma S1P levels were also higher (∼28%; P<0.01) in the obese subjects when compared with the lean controls (Fig. 1C). Next, we performed correlation analysis to examine if a relationship exists between the plasma S1P levels and any clinical indices of the metabolic syndrome. Plasma S1P levels were positively correlated with body fat percentage (Fig. 2A), BMI (Fig. 2B), waist circumference (Fig. 2C), fasting plasma insulin (Fig. 2D), HOMA-IR (Fig. 2E), HbA1c (Fig. 2F), total and LDL cholesterol (Fig. 2G & H). Interestingly, we were unable to detect any correlation between plasma S1P levels and HDL cholesterol, NEFAs, triglycerides or glycerol (data not shown).

### Plasma S1P is Increased with Fasting in Mice

**Figure 3:**
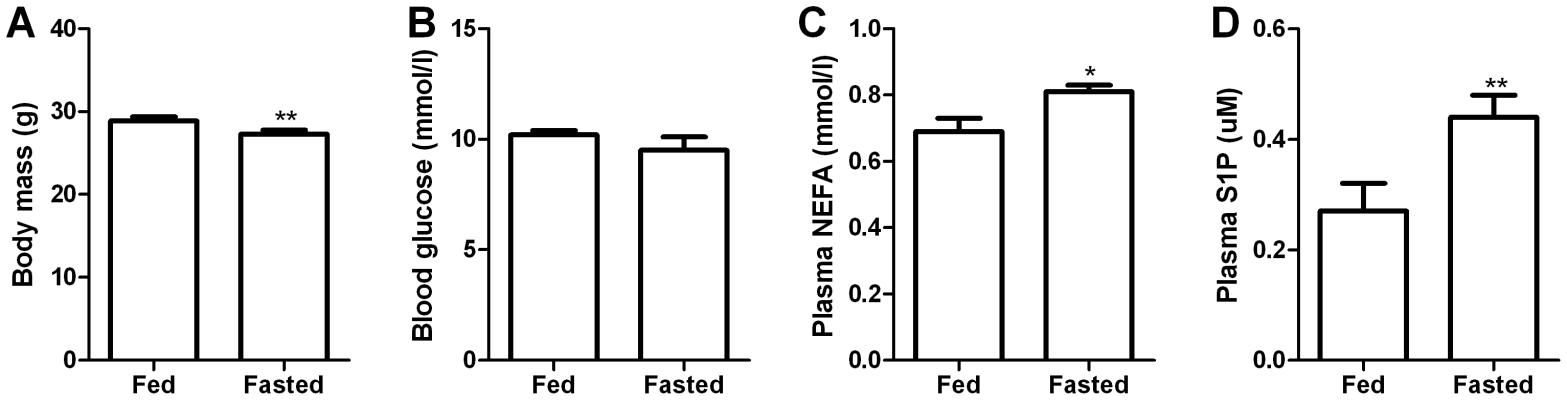
Plasma S1P concentrations in mice are elevated in response to fasting. Changes in body mass (A), blood glucose (B), plasma NEFA (C) and plasma S1P concentrations (D) in response to fasting. Data are mean ± SEM. *P<0.05; **P<0.01, N = 11–12.

Given obesity is associated with increased lipid flux and plasma S1P was elevated in obesity, we next investigated whether an acute fast which increases lipid flux, would also alter plasma S1P concentrations. Plasma S1P levels were measured in both the fed and fasted conditions. Compared to the fed condition, fasting (12 h) resulted in a significant loss of bodyweight (5.5% of starting bodyweight; Fig. 3A) which was paralleled by a significant increase in plasma NEFA levels (Fig. 3B). Interestingly, there was a robust increase (∼60%) in plasma S1P concentration with fasting (Fig. 3C).

## Discussion

Traditionally dyslipidaemia has been characterized by an elevation in plasma triglycerides, total cholesterol and reduction in HDL cholesterol. However, it is becoming increasingly evident that obesity and type 2 diabetes mellitus are characterized by alterations in other circulating lipid species including the bioactive sphingolipid ceramide [4], [8], [14]. Such bioactive lipids may play a more causal mechanistic role in the development of insulin resistance along with other associated complications such as atherosclerosis [8]–[12]. Here we extend those findings by demonstrating that another sphingolipid metabolite, S1P, is significantly elevated in the plasma of obese mice and humans. Furthermore, plasma S1P concentrations in humans positively correlate with clinical indices of metabolic and cardiovascular dysfunction including fasting insulin, HOMA-IR, HbA1c, total and LDL cholesterol, BMI, waist circumference and body fat percentage. We also report that plasma S1P levels in normal healthy mice are robustly increased under acute fasting conditions. Thus it seems that circulating S1P levels are sensitive to changes in metabolic status. Indeed, it is possible that increased lipid flux and/or availability (NEFAs, triglycerides, cholesterol) which are associated with obesity and insulin resistance may govern the production and ultimately the rise in plasma S1P.

It has previously been shown that plasma S1P levels are increased in the genetically obese *ob/ob* mouse [12]. Here we extend upon these findings by showing that in addition to the obese *ob/ob* mouse model, plasma S1P is also increased in HFD fed obese mice and more importantly in obese humans. Furthermore, S1P is positively correlated with clinical indices of the metabolic syndrome. However, whether S1P is simply a biomarker of the metabolic syndrome or whether it plays a mechanistic role in the development of obesity related pathologies such as insulin resistance is not known. This is challenging to resolve especially given the complex and sometimes paradoxical roles S1P plays in mammalian biology [13], [20], [25]. In the context of the metabolic syndrome and cardiovascular disease, S1P has been shown to have pro-survival (anti-apoptotic) [26] and anti-inflammatory actions on hepatocytes [27], kidney podocytes [28], protective actions on the cardiovascular system [29], including improvements in blood flow recovery following ischemia [30], and has been implicated in the metabolically protective actions of adiponectin [31]. S1P has also been shown to have anti-inflammatory actions on macrophages [32]. This may be particularly relevant as obesity is characterized by chronic low-grade adipose tissue inflammation, thought to be predominantly mediated by macrophages that infiltrate obese adipose tissue which secrete pro-inflammatory cytokines [33]. However, in contrast to these findings in macrophages, treatment of adipocytes with S1P has been shown to have pro-inflammatory/pro-thrombotic actions, leading to increased gene expression of plasminogen activator inhibitor-1, TNF-α, interleukin-6, and keratinocyte-derived chemokine [12], thus adding further complexity to its role in obesity and metabolic dysfunction. While S1P has been shown to have many protective actions that improve cell survival under pathological stress settings, these same actions, particularly those related to its effects on anti-apoptotic pathways and angiogenesis have however strongly implicated S1P in the development of cancer [34]. Given obesity is associated with increased prevalence of certain cancers [35], it is therefore difficult to ascertain whether the increase in S1P would be beneficial or detrimental in the setting of obesity. Nonetheless, future research should focus on large population studies to determine if there is a relationship between the incidence of cancer and high circulating S1P concentrations.

The findings of our study show that circulating S1P is elevated in obesity, however, we can only speculate regarding the mechanisms. Recently, studies in mice have shown that S1P regulates glucose stimulated insulin secretion in pancreatic β-cells [36], while its clinically approved therapeutic analogue FTY720 (Fingolimod), which is a S1P receptor agonist, normalizes blood glucose levels in type 2 diabetic *db/db* mice by increasing β-cell mass and circulating insulin levels [37]. S1P has also been shown to increase basal glucose uptake in C2C12 muscle cells [22], however, the consequences of increased plasma S1P on *in vivo* glucose metabolism are not known and whether S1P affects insulin-stimulated glucose uptake is not clear. In addition, our own recent work has shown that FTY720 treatment reduced muscle ceramide, diacylglycerol and triglyceride levels in HFD induced obese mice, ultimately leading to improved whole body glucose homeostasis [38]. In support of the potentially protective role of S1P in obesity, endurance trained humans, who are remarkably insulin sensitive [39], [40], have been shown to have ∼40% higher plasma S1P concentrations than untrained controls [41]. Similarly, one single exercise bout in untrained human subjects, which is a physiological situation that is accompanied by a simultaneous increase in lipid flux [42] (ie. adipose tissue lipolysis, skeletal muscle lipid uptake and oxidation) and insulin sensitivity [43], increases plasma S1P levels by ∼42%, an effect that is accompanied by decreased erythrocyte ceramide levels [41]. This is somewhat similar to our fasting experiment in mice, which like exercise, would be expected to improve insulin sensitivity while also increasing lipid flux [44], resulting in a significant rise in plasma S1P levels (Fig. 3D). Thus it is plausible that an increase in lipid flux which is evident by elevated rates of lipolysis and NEFA release into the circulation, as seen in obesity [45], type 2 diabetes [46], exercise [42] and fasting [47] may result in increased S1P synthesis and secretion into the plasma. In relation to fasting however, previous research in humans has shown that a 10 h overnight fast did not result in altered S1P concentrations when compared to the fed state [48]. This may possibly be explained by the difference in the relative severity of the fast in mice compared to humans. For example, a 10 h fast in humans has relatively minor effects on hepatic glycogen content [49], [50], while in mice, a 12 h fast results in almost complete depletion of hepatic glycogen [44]. Based on the collective results of the abovementioned studies, we speculate that S1P may elicit potentially protective effects by promoting pancreatic β-cell regeneration/survival during the forced compensatory hyperinsulinaemia that is required to maintain euglycaemia in an insulin resistant obese state. Additionally, based on the fact that we have previously shown that the S1P analogue FTY720 prevents muscle lipid accumulation in HFD mice [38], it is possible that elevated plasma S1P may play a role in trying to minimise excessive bioactive lipid build-up in tissues such as liver, pancreatic β-cells and skeletal muscle, an effect most likely mediated by its ability to signal via the S1P receptors [38]. Although the origin of triacylglycerol and diacylglycerol is distinct to that of ceramide, it suggests that alterations in sphingolipid metabolism can have pronounced effects on numerous lipid classes, including those not directly linked to ceramide. Clearly more work is required to further elucidate the biological role of circulating S1P, particularly in the regulation of pancreatic β-cell function and energy metabolism.

While it is well known that the majority (∼54%) of the plasma S1P pool is bound to HDL cholesterol [17], [19], we did not find an association between plasma S1P levels and the total HDL cholesterol fraction (r = −0.128, P = 0.54). In support of this lack of a correlation with HDL, recent studies by Hammad and colleagues have shown that S1P transport in lipoproteins was not limited by the concentration of HDL cholesterol in individual subjects due to the high variability of S1P content in HDL particles [17]. Additionally, the relative ‘per particle’ S1P content was actually highest in the larger VLDL particles [48], thus possibly explaining our positive correlations between plasma S1P with total and LDL but not HDL cholesterol (Fig. 2G & H). It is also worth noting that some of the cardio-protective functions of HDL have been attributed to its high S1P content [51]. The S1P cargo on HDL particles may be cardio-protective due to its cell survival actions on cardiomyocytes and effects on the vascular endothelium that enhance blood flow/angiogenesis [51], [52].

The source of the increased S1P levels in obesity is unclear and requires further investigation. Given the bulk of the circulating S1P pool comes from erythrocytes, leukocytes and vascular endothelial cells [16], [18], there is a distinct possibility that these cells may also be over-secreting this molecule in obesity. However, the possibility remains that the source could be other cells that are more directly affected by obesity such as adipocytes and/or hepatocytes, although it has been reported that hepatocytes do not secrete S1P, and that hepatic derived S1P is of endothelial origin [18]. Furthermore, the increased plasma S1P levels seen in obesity could also potentially result from decreased cellular/tissue S1P degradation by the cleavage enzyme S1P lyase. If this was to be the case, decreased S1P clearance rather than increased secretion could be the reason circulating S1P is increased in obesity.

In conclusion, we demonstrate that plasma S1P is elevated in obese humans and is positively correlated with clinical features of metabolic dysfunction, particularly fasting insulin, HOMA-IR, HbA1c, total and LDL cholesterol, BMI, waist circumference and body fat percentage. Furthermore, circulating S1P is also elevated in HFD induced and genetically obese *ob/ob* mice. Additionally, we also show that an acute period of fasting (12 h) in mice robustly increases plasma S1P levels. Collectively these results show that plasma S1P concentrations are altered in association with metabolic status, possibly via an increase in lipid flux and/or availability. Future research should focus on determining the role that circulating S1P may have on nutrient metabolism and whether it contributes to or alleviates metabolic abnormalities such as insulin resistance and defective glucose homoeostasis.
